# The Use of Buccal Fat Pad Versus Buccal Mucosal Flap in Cleft Patient Palatoplasty—A Literature Review

**DOI:** 10.3390/jcm14093114

**Published:** 2025-04-30

**Authors:** Gabriel Armencea, Gosla Srinivas Reddy, Simion Bran, Alexandru Bereanu, Damaris Anton, Florin Onișor, Cristian-Mihail Dinu, Alexandra Denisa Papuc, Sebastian Stoia, Tiberiu Tamaș, Mihaela-Felicia Băciuț

**Affiliations:** 1 Department of Oral and Maxillofacial Surgery and Radiology, Faculty of Dental Medicine, “Iuliu Hațieganu” University of Medicine and Pharmacy, TEAM Group Project, 400012 Cluj-Napoca, Romania; garmencea@gmail.com (G.A.); alexandru.bereanu@yahoo.com (A.B.); florin.onisor@umfcluj.ro (F.O.); dinu_christian@yahoo.com (C.-M.D.); dr.alemuresan@gmail.com (A.D.P.); stoia_sebi@yahoo.com (S.S.); tiberiu.tamas@umfcluj.ro (T.T.); mbaciut@yahoo.com (M.-F.B.); 2 G.S.R. Hospital, Institute of Cranio-Maxillofacial and Facial Plastic Surgery, Vinay Nagar Colony, Saidabad, Hyderabad 500059, India; goslareddy@gmail.com; 3 Department of Periodontology, Faculty of Dental Medicine, “Iuliu Hațieganu” University of Medicine and Pharmacy, 400012 Cluj-Napoca, Romania; damaris.anton@elearn.umfcluj.ro

**Keywords:** buccal fat pad flap, buccal mucosal flap, palatoplasty

## Abstract

The buccal fat pad and buccal mucosa are anatomical structures closely related to palatal clefts which can provide additional tissues for defect reconstruction which is crucial for practitioners aiming to lessen the need for additional corrective surgeries in patients with cleft palates and to lower the rates of residual palatal fistulae. **Objectives**: Aims to explore the recent scientific data available on the applications and outcomes of two surgical techniques involving the buccal fat pad and buccal mucosal flap in primary and secondary palatoplasty. **Methods**: The analyzed articles published between 2020 and 2025 from PubMed, Web of Science, and Scopus. The search strategy included terms related to buccal fat pad flaps, buccal mucosal flaps, and cleft palate repair. **Results**: After performing the search, including eligible articles and removing duplicates, 15 articles were included in this review. Eight studies explored the effectiveness of buccal fat pad or buccal mucosal flap during primary palatoplasty and seven studies for secondary palatoplasty. The articles included in this review provide insights on the usefulness of buccal fat pad flaps and buccal mucosal flaps in primary and secondary palatoplasty. **Conclusions**: The buccal fat pad and buccal mucosal flaps are highly effective in secondary palatoplasty, particularly for velopharyngeal dysfunction and fistula closure. In primary palatoplasty, the buccal fat pad flap aids mucosal healing, reduces complications, and improves speech, while the buccal mucosal flap is beneficial for wide palatal defects.

## 1. Introduction

### 1.1. Anatomical and Physiological Considerations

The use of the buccal fat pad (BFP) and buccal myomucosal flap in cleft palate surgery requires a comprehensive understanding of the facial anatomy. The buccal fat pad is a complex structure related to important anatomical structures such as the facial nerve, parotid gland, and masticatory muscles. Moreover, it has great importance in facial aesthetics and contour [1]. BFP is known as a practical and useful flap for treating many defects of the oral cavity, including congenital palatal clefts, but is also used in other intraoral defects like osteonecrosis, tumours, and oro-antral communications. The benefits of using this flap derive from its rich blood supply and proximity to various defects of the oral cavity [2] and the size and availability of fat tissue for small to medium defect reconstruction. The buccal myomucosal flap (BMMF) was initially introduced as an adjunct to Furlow palatoplasty by Fisher and Mann and has been around for over two decades offering effective solutions for complex cases and improving patient outcomes in speech and quality of life [3]. BMMF, also known as buccinator flap, can be raised in many various forms, including as a myomucosal flap, which is particularly beneficial for reconstructing mucosal defects associated with cleft palates [4] in primary and secondary reconstruction.

### 1.2. Surgical Techniques and Outcomes

In the available literature there are a variety of methods involving the buccal myomucosal flap and buccal fat pad regarding surgical techniques such as flap harvesting and incision designs [5]. There are many discussions about consequences including flap necrosis, infection rates and aesthetic considerations even though studies show positive outcomes in terms of tissue integration and aesthetic appearance [6]. The requirement for surgical guidelines is highlighted by the fact that different practitioners have different results with these approaches.

### 1.3. Patient Outcomes and Quality of Life

After cleft palate surgery, patient satisfaction and quality of life are of major importance. According to the available research, the BFP and BMMF can improve speech outcomes, lower the need for follow-up procedures, and increase confidence and general satisfaction amongst patients and their families. Also, the unilateral myomucosal buccinator flap appears to be an effective surgical option for treating velopharyngeal insufficiency, leading to significant improvements in speech intelligibility, resonance, and velopharyngeal closure in the majority of patients [7].

### 1.4. Complication Management After Cleft Palate Surgery

Palatal fistulae is a common complication that can occur after cleft palate repair. The incidence of recurrence after the initial closure of palatal fistulas is notably high. Many surgical techniques have been proposed to address this issue, but achieving a permanent closure can be challenging. The use of a buccal myomucosal flap for the closure of posterior palatal fistulas demonstrated high efficacy and safety, making it a valuable technique in surgical practice for patients with cleft palate repair complications [8]. Other complications mentioned in the literature are buccal swelling, infections, parotid duct stenosis, difficulties in molar eruption, and some unusual complications such as the herniation of the buccal fat at the donor site. Despite all these complications, the buccal flap is a useful tool of cleft surgery with minimum donor site morbidity and complications [9].

### 1.5. Buccal Fat Pad or Buccal Mucosal Flap

Some authors prefer the buccal fat pad over buccal mucosal flap due to its rich vascularization and the fact that it is easily accessible for intraoral reconstruction. It can effectively fill and line denuded (exposed) areas, enhancing vascularity and potentially preventing significant wound contraction; also, the buccal fat pad is quicker to apply and minimizes donor-site morbidity and surgical time. Moreover, there is no report in changes in the cheek volume of children as only a small part of the lobe is used for reconstruction. Some authors encourage the inclusion of the buccal fat pad flap technique in the surgery for challenging cleft palates that have a wide transverse diastasis, emphasizing its role in reinforcing high-tension areas and reducing the risk of fistulae formation [10,11].

This literature review aims to explore the recent scientific data available on the application and outcome of two surgical techniques involving the buccal fat pad and buccal mucosal flap in primary and secondary palatoplasty. Although various individual studies have investigated the surgical outcomes of BFP and BMMF, to the best of our knowledge, no comprehensive synthesis critically compares their indications, surgical outcomes, complications, and long-term results in both primary and secondary palatoplasty settings. The authors believe it is mandatory to have an overview on the existing literature in order to improve surgical methods that directly enhance patient care in cleft palate surgery. We aim to investigate the benefits of using these two anatomical structures that give extra tissue for palate closure since they are crucial for practitioners who wish to lessen the need for additional corrective surgery in patients with cleft palate and to lower the rate of residual palatal fistulae.

## 2. Materials and Methods

The available literature search was performed using three known databases through the institutional access provided by the library of the “Iuliu Hațieganu” University of Medicine and Pharmacy. Two different authors conducted the searches based on strategies created for each database using frequent keywords that can be found in the existing literature (Table 1). The results obtained (234) were imported in a platform, https://www.rayyan.ai/ version 1 (accessed on 1 February 2025) (Cambridge, MA, USA), where duplicate elimination was performed. The selecting process of relevant studies was carried out based on inclusion and exclusion criteria (Table 2). Each author completed the screening by reviewing the full text of the article. Only articles that fulfilled all the inclusion criteria were considered eligible; therefore, each full-text article was obtained to determine whether it would be included or not in the review. All eligible articles were rigorously evaluated by each author, and a total of 15 studies were finally included in the literature review.

## 3. Results

**Figure 1:**
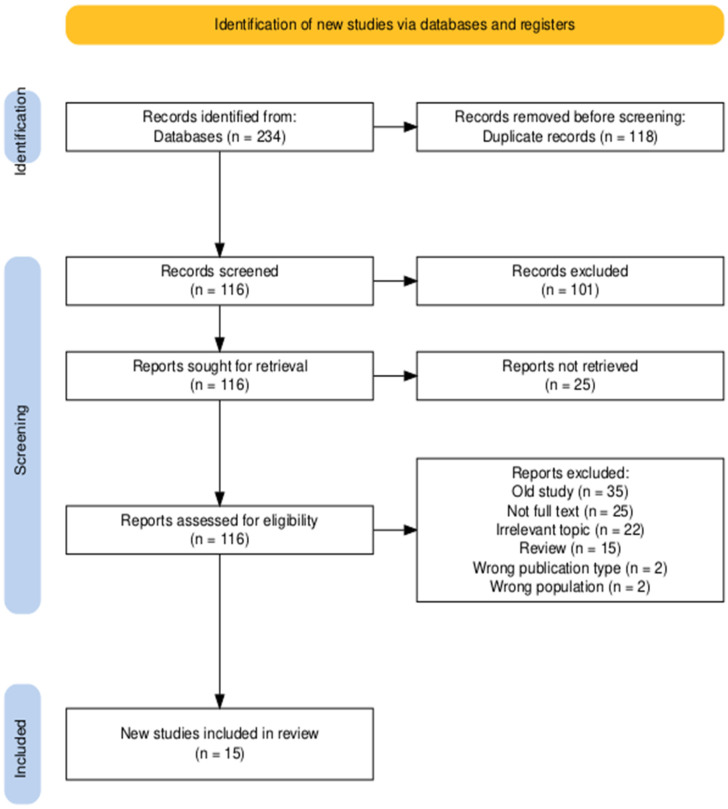
PRISMA 2020 flow diagram for new systematic reviews which included the searches of databases and registers only [12].

A total of 234 records were obtained after database searching, and 118 duplicates were removed. The title and abstract screening eliminated 101 publications due to excluding reasons such as old studies, irrelevant topic, unavailable full text, reviews, or meta-analysis. In the end, 15 studies were considered eligible and were included in the review. The identification and selecting process is shown in the following PRISMA diagram (Figure 1).

The studies included in this review were divided into two categories based on the surgery timing of cleft palate: primary and secondary palatoplasty. There are eight studies that explore the effectiveness of buccal fat pad or buccal mucosal flap during primary palatoplasty and the other seven studies for secondary palatoplasty. To facilitate thematic analysis, the studies were further categorized according to the primary outcome focus: anatomical modifications, postoperative speech and functional results, rates and types of complications (especially fistula formation), and donor site morbidity (Table 3 and Table 4).

## 4. Discussion

Seven observational studies were found to evaluate the application of buccal fat pad in primary cleft palate surgery, from which three are prospective studies [13,16,17], one is a preliminary research [14], another is a comparative study [15], one is a cohort study [18], and one study follows a retrospective design [20]. Khan et al.’s study [13] explored the healing and mucolization of the buccal fat pad graft in cleft palate surgery. Kotlarek et al. [14] investigated the impact of BFP flap on velopharyngeal and levator veli palatini muscle function post primary cleft surgery. Chi-Chin Lo et al.’s study [15] and the retrospective study [20] explored the maxillary development, scar formation, palatal contraction, and fistula complications after using the BFP technique. Another study by Haensler et al. [17] evaluated the outcome of speech intelligibility and resonance after using buccal flap technique. In another study, Natsir-Kalla DS et al. [16] underlined the effectiveness of BFP in reducing the risk of blood loss during primary cleft palate surgery. A cohort study [18] assessed the maxillary arch dimension in primary dentition cleft palate patients who underwent two-flap palatoplasty (TFP) and Furlow palatoplasty with the buccal myomucosal flap (FPBF). Only one randomized clinical trial [19] investigated the efficacy of buccinator myomucosal flap in primary palatoplasty predicting the risk of post-surgical complications and velopharyngeal insufficiency.

Khan I. et al.’s prospective study [13] focuses on the use of the buccal fat pad (BFP) as a pedicled graft to cover lateral palatal defects in cleft palate repair surgeries. The research aimed to compare the outcomes of incorporating BFP versus traditional methods for defect closure post-cleft palate repair. The study involved 42 patients undergoing cleft palate repair, both females and males aged between 8 and 14 months, from which 21 received BFP as an additional step, while 21 underwent conventional cleft palate repair. The BFP surgical technique offers a detailed process for effectively covering lateral palatal defects in cleft palate repair, emphasizing gentle handling of the fat pad to promote successful outcomes with minimal complications. Complications were minimal with 95.2% of patients experiencing no issues from donor or recipient sites. By the second week post-repair, 95.2% of BFP patients achieved complete mucolization compared to only 4.8% in the conventional repair group. Statistical tests indicated a significant association between the study group and mucolization status on days 14 and 21 (*p* < 0.001). The BFP emerges as a valuable source of vascularized tissue for covering hard palate bones post primary cleft repair due to its benefits: ease of harvest, faster epithelialization, and low complication rates.

A preliminary study from Katelyn J. Kotlarek et al. [14] investigated the impact of using a pedicled buccal fat pad (BFP) flap in primary palatoplasty on the levator veli palatini (LVP) muscle and surrounding velopharyngeal anatomy, and it utilized MRI to analyze how the BFP flap affects LVP and VP anatomy postoperatively, revealing significant differences in various measurements among the three participant groups. The BFP flap could prevent LVP anterior migration resulting in a more flexible and thicker palate. Participants with BFP flap showed longer effective velar length compared to other groups which could impact VPI and palate revision. Also, effective VP ratio differences were observed between groups, with the BFP group having a larger ratio due to increased effective velar length. Compared to traditional methods without added tissue, the BFP technique demonstrated notable anatomical variations.

The comparative outcome study by Chi-Chin Lo et al. [15] assessed the transverse maxillary development, through cone beam computed tomographic scans, on patients who underwent primary palatoplasty with pedicled buccal fat flaps and compared the outcomes against the data from patients who received a similar palatoplasty with Surgicel coverage of the lateral raw surfaces and patients who had no deformity of the secondary palate (nonpalatoplasty group). A total of 78 patients with unilateral cleft deformity were analyzed. The buccal fat group had wider maxillary dimensions than the Surgicel group, and the nonpalatoplasty group had larger sagittal palatal dimensions. Moreover, buccal fat flaps reduced posterior maxillary constriction, and scar formation was minimized through buccal fat usage.

Natsir-Kalla DS et al. [16] published a prospective study that investigated the risk of complications during palatoplasty using BFP, especially intraoperative and early postoperatively blood loss. This complication is a critical concern during palatoplasty, particularly in younger patients. This study aimed to identify factors affecting blood loss during palatoplasty using the DOZ Furlow technique with BFP grafts. The study addresses a gap in the literature regarding intraoperative blood loss during palatoplasty. While previous studies have discussed blood loss, there is a lack of consensus on the relationship between patient-related factors and blood loss during surgery. The findings suggest that older children are at a higher risk for increased intraoperative blood loss, particularly in rural areas. This observation is supported by existing literature that indicates age and weight are critical factors for surgical outcomes [28,29,30].

Another prospective study by Abigail E. Haenssler et al. [17] evaluated the effectiveness of the buccal flap technique in enhancing anatomical structures that are critical for speech and velopharyngeal (VP) closure. The results from MRI analysis indicate that the buccal flap group have a significantly longer effective velar length and a larger effective VP ratio compared to the non-cleft group. This suggests that the buccal flap technique successfully repositions the levator veli palatini muscle, enhancing speech function, specifically intelligibility and resonance.

Effects of two-flap palatoplasty versus Furlow palatoplasty with buccal myomucosal flap on maxillary arch dimensions in patients with cleft palate at the primary dentition stage: a cohort study [18] assessed the efficacy of the two-flap palatoplasty (TFP) compared to the Furlow palatoplasty with the buccal myomucosal flap (FPBF) in children with primary dentition with cleft palate on their maxillary arch dimensions. The sample size consisted of 28 subjects between 5 and 6 years of age, of which 10 were children without a cleft palate which were included in the control group, 9 subjects were with a cleft palate treated with TFP and 9 subjects were with a cleft palate treated with FPBF. The authors concluded that both the FPBF and TFP resulted in a greater symmetry of the maxillary arches compared to the control group; however, the FPBF provided greater maxillary arch dimensions as compared to the TFP.

A Novel Technique Predicting Velopharyngeal Insufficiency Risk in Newborns Following Primary Cleft Repair [19] assessed the buccinator myomucosal flap (BMMF) compared to the Bardach two-flap effectiveness in primary cleft palate plasty by analyzing palatal length and fistula formation through a randomized clinical trial. The sample size consisted of 46 subjects between 9 and 18 months of age. The subjects were later divided in two groups based on the palatoplasty techniques being compared and examined at 1 week, 3 months, and 6 months for fistula formation. The authors concluded that the BMF is a straightforward technique providing additional flexibility allowing for its simple manipulation in closing wider palatal clefts. The BMF offered a more favourable prognosis while addressing velopharyngeal insufficiency as compared to the Bardach two-flap technique. In conclusion, 1 of the 23 BMF subjects presented with a fistula, whereas 4 of the 23 Bardach two-flap subjects presented with a fistula. Although the fistulation rate was lower in the BMF, there was not a statistically significant difference.

The retrospective study titled “Filling the Void: Use of Interpositional Buccal Fat Pad to Decrease Palatal Contraction and Fistula Formation” [20] explored the efficacy of using the BFP flap to address voids after muscle transposition during cleft palate surgery. Through a retrospective analysis of patients under 3 years of age who had primary palatoplasty, the findings indicate a decrease in palatal shortening, lower fistula rates, and positive speech outcomes with buccal fat pad flap utilization. The BFP flap technique proves valuable in challenging cleft repairs by reducing the need for secondary interventions for speech improvement and providing long effects in maintaining palatal length.

*THE USE AND OUTCOMES OF BUCCAL FAT PAD AND BUCCAL MYOMUCOSAL FLAP IN SECONDARY PALATOPLASTY*

Seven observational studies explored the use of buccal fat pad and buccal myomucosal flap in secondary cleft surgery. There are three retrospective studies [21,25,26] from which one investigates the buccal fat pad in secondary cleft repair [21] and the other two [25,26] explore the application of buccal myomucosal flap in velopharyngeal dysfunction management. Other two studies are designed as prospective [22,23] and evaluated buccal flap techniques in secondary palatoplasty. One cohort study [27] also explored the effectiveness of secondary Furlow palatoplasty using the buccal flap. There was one case report [24] in which a patient was treated for a wide palatal fistula with both buccal fat pad transfer and buccinator myomucosal flap.

In a retrospective study on buccal fat pad in cleft palate repair, institutional experience of 27 cases [21] used the buccal fat pad in cleft lip and palate reconstruction with the aim to use and evaluate surgical techniques and outcomes. It examined 27 cases over three years, highlighting the efficacy of BFP in primary and secondary palatoplasty. The results indicate favourable healing and speech evaluation in the majority of patients, emphasizing the value of BFP in moderate defects. Among the 25 secondary cases, 7 patients were treated for velopharyngeal insufficiency, while 18 patients underwent fistula closure surgery. BFP was effective in promoting tension-free palatal closure and reducing scar formation and was particularly useful for fistula closure in secondary palatoplasty. BFP, in conjunction with other flap techniques described in the study (V-Y pushback technique, Furlow technique), proves to be effective for both primary and secondary cleft surgery, showing a high success rate and minimal complications.

Ahmed E. et al.’s prospective study [22] aimed to evaluate the effectiveness of a modified surgical technique called buccinator re-repair combined with intralveolar veloplasty for treating velopharyngeal insufficiency (VPI) in patients who underwent previously palatoplasty. The findings indicate significant improvements in various speech parameters, including hypernasality, nasal emission, facial grimace, weak consonants, and speech intelligibility. Endoscopic and videofluoroscopic exams showed improvements in total and functional velar length, closure ratio, velopharyngeal gap at closure, palatal thickness, convexity, and mobility. The study concluded that the used technique is effective and safe for managing challenging cases of post-palatoplasty VPI.

The authors of another study performed palatal lengthening by double opposing buccal flaps for the surgical correction of velopharyngeal insufficiency in cleft patients [23] and assessed the effectiveness of the double opposing buccal flap in treating velopharyngeal insufficiency by lengthening the soft palate. The prospective study included a sample size of 50 subjects (14 adults, 12 adolescents, and 24 children). The subjects had undergone the double opposing buccinator myomucosal flap following the assessment of any postoperative complications. None of the subjects following the surgical treatment presented with hyponasal speech postoperatively. The authors concluded that the double buccal flap technique is a viable and reliable surgical treatment option for addressing velopharyngeal insufficiency in cleft patients of all ages.

Through the use of a buccinator myomucosal flap and bilateral pedicled buccal fat pad transfer in wide palatal fistula repair, a case report [24] assessed a 5-year-old male subject with a cleft lip and a wide cleft palate. The subject was treated with a pharyngeal flap; however, a large V-shaped palatal defected followed, associated with velopharyngeal insufficiency and sever hypernasality. The revision of the palatal fistula was performed utilizing a pedicled buccal fat pad, palatal lengthening with a buccinator myomucosal flap, and a sphincter pharyngoplasty to address the wide palatal defect. After 2 weeks, the palate healed with mucolization, and after 4 years, no further complications arouse. Therefore, it was concluded that the revision surgery utilizing the aforementioned flaps was considered a viable option for the closure of wide palatal fistulas, as well as addressing the velopharyngeal insufficiency arising from the primary palate repair.

The retrospective study titled “Buccal Myomucosal Flap Repair for Velopharyngeal Dysfunction” [25] assessed the effects of efficacy of the buccal myomucosal flaps in treating velopharyngeal insufficiency following primary palatal repair. The sample size consisted of 25 subjects with a median 8.8 years of age. The authors concluded that there was a significant improvement in velar closing, and speech scores postoperatively, so velopharyngeal insufficiency following primary palatal repair can be treated utilizing buccal myomucosal flaps.

Another study titled “Comparative Effectiveness of Secondary Furlow and Buccal Myomucosal Flap Lengthening to Treat Velopharyngeal Insufficiency” followed a retrospective design [26] to assess the effects of Secondary Furlow and buccal myomucosal flaps (BMMF) in treating velopharyngeal insufficiency by evaluating the following parameters: palatal lengthening and levator veli palatini muscle positioning. The sample size consisted of 32 pediatric subjects where 12 subjects received a secondary Furlow treatment, and 20 subjects received the buccal myomucosal flap treatment. The authors concluded that both the Furlow and BMMF treatments increased palatal length, resulting in similar improvements in hypernasality resulting in favourable speech results.

The effectiveness of secondary Furlow palatoplasty with buccal myomucosal flap in the correction of velopharyngeal insufficiency in patients with cleft palate [27] assessed through a cohort study the efficacy of the secondary Furlow palatoplasty with the buccal myomucosal flap (FPBF) in addressing the velopharyngeal insufficiency in cleft palate subjects who had undergone primary palatoplasty using the two-flap palatoplasty (TFP). The sample size consisted of 23 subjects between 4 and 8 years of age. The authors concluded the secondary Furlow palatoplasty with the buccal myomucosal flap following the primary palate repair was able to successfully ameliorate hypernasality and improve speech intelligibility with statistically significant improvements in the nasopharyngoscopic scores.

The predominance of low-quality and small-scale studies, significant variety in surgical techniques, and possibility of donor site morbidity contribute to the scarcity of the available literature on the use of the buccal mucosal flap during primary palatoplasty.

Firstly, the reduced sample sizes and retrospective study designs are frequently encountered because cleft patients’ treatments are performed in specialized centres where many have a limited number of patients, making it difficult to construct a large-scale prospective, high-quality research.

Secondly, the significant variability in surgical technique, as well as flap design which is needed to personalize the treatment dependant on the patient’s anatomy, complicates the possibilities in achieving a standardized protocol for research purposes.

Lastly, while the reported buccal mucosal flaps may offer minimal donor site morbidity, clinicians may be hesitant to rely on such a flap due to the possible under-reported complications such as donor site contracture, sensory disturbances, or an impairment of oral function, thus leading to a deficit of the available literature on the use of the buccal mucosal flap as a primary treatment option.

## 5. Conclusions

The reviewed studies emphasize the benefits of using BFP in primary cleft palate repair, particularly in promoting mucosal healing, reducing the risk of complications and enhancing functional outcomes such as speech and maxillary development. Buccal myomucosal flap can also be used in primary palatoplasty with good results in managing challenging and wide palatal defects. Both buccal fat pad and buccal myomucosal flap show high success rates in secondary palatoplasty, particularly for velopharyngeal dysfunction and fistula closure. Significant improvements in speech intelligibility after secondary palatoplasty were observed in multiple studies. Despite the encouraging findings, the overall quality of evidence is limited by the predominance of retrospective designs, small sample sizes, and absence of control groups in most studies. Only one randomized controlled trial [19] attempted to standardize confounding factors such as cleft severity, surgical technique, and timing.

Further studies should compare the use of BFP in primary surgery on the midline, on top of the nasal layer closure; for wide palatal clefts, most of the studies are carried out on the use of the BFP on the raw surfaces of the alveolar processes together with Bardach technique. For secondary surgery and treatment of oro-nasal fistulae BFP and BMMF should be studied for midline closure with/without a nasal layer closure.

The suggestion to reserve BFP or BMMF exclusively for secondary procedures, in case oro-nasal fistulae would occur, is debatable because a correct use and indication for these flaps in primary surgery may proactively reduce postoperative complications.

Future research should focus on randomized controlled trials to compare more efficiently different surgical techniques and provide standard surgical protocols for practitioners.

## Figures and Tables

**Table 1:** Search strategies.

Database	Search Strategy	Results
PubMed	(“buccal fat pad” OR “buccal fat pad flap” OR “buccal myomucosal flap”) AND (“cleft palate” OR “cleft surgery” OR “cleft palate surgery” OR “primary palatal surgery” OR “secondary palatal surgery” OR “palatoplasty”)	62
Scopus	TITLE-ABS-KEY((“buccal fat pad” OR “buccal myomucosal flap”) AND (“cleft palate” OR “cleft palate surgery” OR “palatoplasty” OR “congenital cleft” OR “primary palatal surgery” OR “secondary palatal surgery”))	79
Web of Science	(((TS = (buccal fat pad)) OR TS = (buccal myomucosal flap)) AND TS = (cleft palate))	93

**Table 2:** Inclusion and exclusion criteria.

Inclusion Criteria	Exclusion Criteria
Relevant topic Studies from the last 5 years (2020–2025) Studies published in English	Reviews, systematic reviews, meta-analysis Studies published before 2020 Animal model studies Other foreign languages

**Table 3:** Studies assessing primary palatoplasty.

Reference	Publication Year	Study Design and Population	Conclusions
The Application of Buccal Fat Pad to Cover Lateral Palatal Defect Causes Early Mucolization [13]	2021	Prospective study 42 cleft palate patients	-buccal fat pad (BFP) effectively covers hard palate defects -BFP is easy to harvest with a low learning curve -epithelization rate is faster than conventional methods -minimal complications rates observed with BFP usage
A Preliminary Study of Anatomical Changes Following the Use of a Pedicled Buccal Fat Pad Flap During Primary Palatoplasty [14]	2021	Preliminary research study 15 children aged 3–7 years divided in 3 groups: 5 patients with cleft palate and/or lip who underwent primary palatoplasty with BFP flap, 5 patients received a traditional repair without addition of any tissue and 5 healthy non-cleft patients	-the pedicled BFP flap creates a longer velum -increased distance between posterior hard palate and levator veli palatini (LVP) muscle observed -larger effective velopharyngeal ratio compared to traditional techniques
Favourable Transverse Maxillary Development after Covering the Lateral Raw Surfaces with Buccal Fat Flaps in Modified Furlow Palatoplasty: A Three-Dimensional Imaging-Assisted Long-Term Comparative Outcome Study [15]	2022	Comparative study 22 patients with unilateral cleft lip, alveolus and palate in buccal fat flap group and 32 patients in Surgicel group	-covering lateral surfaces with buccal fat flaps reduces maxillary constriction -buccal fat flaps improve posterior transverse maxillary development -there was no increased complication rates observed with buccal fat flaps
Influence of patient-related factors on intraoperative blood loss during double opposing Z-plasty Furlow palatoplasty and buccal fat pad coverage: A prospective study [16]	2022	Prospective study 109 patients treated with DOZ Furlow palatoplasty and BFP graft	-DOZ Furlow palatoplasty with BFP graft is safe -higher weight increases intraoperative blood loss -longer operation time also increases blood loss -operate at an earlier age to reduce blood loss
Anatomical and Physiological Changes Following Primary Palatoplasty Using “The Buccal Flap Approach” [17]	2023	Prospective study 30 adult male patients divided into 2 groups: 15 males born with Veau type 3 or 4 and 15 healthy adults with no history of cleft palate	-the use of the buccal flap with DOZ palatoplasty had favourable soft tissue outcomes for adults with Veau type 3 and 4 clefts -the effective velar length was statistically longer in the buccal flap group compared to the control group
Effects of two-flap palatoplasty versus Furlow palatoplasty with buccal myomucosal flap on maxillary arch dimensions in patients with cleft palate at the primary dentition stage: a cohort study [18]	2023	Cohort 28 Participants	-Furlow palatoplasty with buccal myomucosal flap resulted in better maxillary arch dimensions in patients with cleft palate at the primary dentition stage.
A novel technique predicting velopharyngeal insufficiency risk in newborns following primary cleft repair. A randomized clinical trial comparing buccinator flap and Bardach two-flap palatoplasty [19]	2024	Randomized clinical trial 46 Participants	-reliable technique for primary management of cleft palate patients. -simple and easy technique with low donor site morbidity and low intra- and post-operative complications. -ability to be rotated and advanced in many directions with many patterns of orientation that help in closing wide palatal defects. -in primary repair of soft palate increases the palatal length. -velopharyngeal insufficiency prognosis is much better with BMF than with the Bardach two-flap palatoplasty technique.
Filling the Void: Use of the Interpositional Buccal Fat Pad to Decrease Palatal Contraction and Fistula Formation [20]	2020	Retrospective study 53 patients under age 3 who underwent primary palatoplasty utilizing a medially placed BFPF (buccal fat pad flap)	-BFPF improves cleft palate repair outcomes, reduces fistula formation and maintains palatal length -early results indicate excellent durability in cleft repairs

**Table 4:** Studies assessing secondary palatoplasty.

Reference	Publication Year	Study Design and Population	Conclusions
Buccal fat pad in cleft palate repair- An institutional experience of 27 cases [21]	2020	Retrospective study 27 cases of cleft lip and palate	-BFP is effective for cleft palate repair -BFP has high success rates with minimal morbidity
Buccinator Re-Repair (Bs + Re: IVVP): A Combined Procedure to Maximize the Palate Form and Function in Difficult VPI Cases [22]	2020	Prospective study 30 cases who had Bs + Re: IVVP (buccinator flaps’ lengthening and palate re-repair with radical intravelar veloplasty)	-buccinator re-repair is effective for VPI (velopharyngeal insufficiency) -significant improvement in speech postoperatively
Palatal lengthening by double opposing buccal flaps for surgical correction of velopharyngeal insufficiency in cleft patients [23]	2020	Prospective Study 50 Participants	-buccinator myomucosal flap can be used as an effective and safe method for lengthening the palate to correct VPI in patients of all ages. It can therefore be used as an alternative to Furlow Z plasty, before attempting potentially complicated procedures like pharyngoplasty
Use of a buccinator myomucosal flap and bilateral pedicled buccal fat pad transfer in wide palatal fistula repair: a case report [24]	2021	Case Report 1 Participant	-combination of a bilateral buccinator myomucosal flap and pedicled BFP transfer was a feasible and cost-effective technique. -The patient required long-term follow-up to monitor his speech development and palate growth. -Therefore, a posteriorly based buccinator myomucosal flap might be considered to close large ONFs and improve VPI in young patients with cleft palate.
Buccal Myomucosal Flap Repair for Velopharyngeal Dysfunction [25]	2022	Retrospective Study 25 participants	-Secondary palatoplasty incorporating buccal myomucosal flaps is a safe and effective option for the treatment of VPD, improving postoperative speech outcomes.
Comparative Effectiveness of Secondary Furlow and Buccal Myomucosal Flap Lengthening to Treat Velopharyngeal Insufficiency [26]	2023	Retrospective Study 32 Participants	-both procedures significantly increased velar length and effective velar length and decreased hypernasality by two scalar points.
Effectiveness of secondary furlow palatoplasty with buccal myomucosal flap in correction of velopharyngeal insufficiency in patients with cleft palate [27]	2024	Cohort 23 Participants	-BMMF with Furlow palatoplasty was successful in improving hypernasality, speech intelligibility, and nasopharyngoscopic scores in patients with cleft palate. -might be a surgical procedure with noticeable benefits while treating patients suffering from VPI after cleft palate repair.

## Abbreviations

BFP	Buccal Fat Pad
BMMF	Buccal Myomucosal Flap
LVP	Levator Veli Palatini
DOZ	Double Opposing Z-plasty
BMF	Buccal Mucosal Flap
BFPF	Buccal Fat Pad Flap
VPI	Velopharyngeal Insufficiency
VP	Velopharyngeal
ONF	Oro-nasal Fistulas
VPD	Velopharyngeal Dysfunction
TFP	Two-Flap Palatoplasty
